# Exploring Associations Between COVID-19 Bivalent Vaccines and Their Related Adverse Events: A Correlational Study

**DOI:** 10.21203/rs.3.rs-6152825/v1

**Published:** 2025-03-10

**Authors:** Yiming Li, Wei Tao, Sori K Lundin, Zenan Sun, Yifang Dang, Yong Chen, Cui Tao

**Affiliations:** The University of Texas Health Science Center at Houston; The University of Texas Health Science Center at Houston; The University of Texas Health Science Center at Houston; The University of Texas Health Science Center at Houston; The University of Texas Health Science Center at Houston; University of Pennsylvania; Mayo Clinic

**Keywords:** Adverse event, concept normalization, correlation analysis, COVID-19, COVID-19 Bivalent vaccines, COVID-19 vaccines, natural language processing, vaccine safety monitoring, VAERS

## Abstract

**Introduction:**

COVID-19, a global health crisis, prompted rapid vaccine development. Bivalent vaccines by Pfizer-BioNTech and Moderna target both original and Omicron strains. Despite vaccination efforts, the persistence of adverse events underscores the importance of ongoing safety monitoring.

**Method:**

We collected COVID-19 bivalent vaccine adverse event reports from the Vaccine Adverse Event Reporting System (VAERS) (September 15, 2022, to September 1, 2023). The Medical Dictionary for Regulatory Activities (MedDRA) categorized reported symptoms into corresponding concepts. Statistical analyses included zero-truncated Poisson regression for estimating symptom rates, logistic regression for identifying risk factors, and Spearman correlation for assessing non-parametric relationships between adverse events in different System Organ Classes (SOCs).

**Results:**

Our analysis of COVID-19 bivalent vaccine adverse events identified significant correlations between SOCs. Notably, a positive correlation (ρ = 0.3) linked Infections (SOC 1) with Respiratory disorders (SOC 13), while a negative correlation (ρ=−0.3) connected Investigations (SOC 23) with Injury (SOC 24). Zero-truncated Poisson modeling estimated an average of 6.179 symptoms per individual, varying by age, gender, and vaccine. Gender and age significantly influence reported adverse events associated with COVID-19 bivalent vaccines. Females generally experienced higher odds for most SOCs compared to males, except for cardiac and product-related issues. Older adults showed a higher likelihood of symptoms across various SOCs, while children and adolescents had distinct susceptibility patterns. Additionally, Moderna and Pfizer vaccines were associated with different SOCs, with Moderna linked mainly to immune and skin disorders and Pfizer associated primarily with cardiac and metabolic issues.

**Conclusion:**

In conclusion, our study aligned self-reported items with widely-accepted medical terminology and rigorously analyzed adverse events following COVID-19 bivalent vaccine using advanced statistical methods. The significant findings provide crucial insights, guiding evidence-based strategies and interventions for vaccine safety.

## Introduction

COVID-19, short for Coronavirus Disease 2019, is an emerging global health crisis caused by the coronavirus. The impact of COVID-19 has led to significant disruptions in economies, strained healthcare systems, and altered the daily lives of billions [1].It has caused more than 773 million cases and nearly 7 million deaths till December 2023 [2]. In particular, approximately 76% of all recorded deaths are among individuals aged 65 and above [3]. The virus spreads quickly due to its high transmissibility, the most common ways of infection are through respiratory droplets or by touching the eyes, nose, or mouth with hands carrying SARS-CoV-2 particles [4]. Symptoms of COVID-19 ranged from fevers and sore throat to trouble breathing, which typically last about 2 to 14 days [5]. Patients might experience long-haul symptoms, commonly referred to as long COVID, which can persist for more than 12 weeks after injection [6].

Vaccines play a pivotal role in managing and ultimately stopping the pandemic by preparing the body’s immune system to recognize and kill the virus [7], [8], [9], [10], [11], [12]. The development of bivalent vaccines by Pfizer-BioNTech and Moderna represents a substantial step forward in addressing both the initial COVID-19 virus and the Omicron variants BA.4 and BA.5. [13]. These bivalent vaccines demonstrated a 37% higher effectiveness compared to older vaccine in lowering the chances of severe COVID-19. All patients, regardless of their booster history or age, can derive benefits from these vaccines [14]. The Centers for Disease Control and Prevention (CDC) has also advised that individuals aged 5 years and older should receive at least one dose of the COVID-19 vaccine to safeguard against severe illness [13].

As the world embarked on an unprecedented vaccination campaign against COVID-19, the focus on vaccine safety became paramount. Adverse events following immunization (AEFIs) have been a subject of extensive scrutiny and discussion [15]. These events encompass a spectrum of reactions that occur after receiving a COVID-19 vaccine, ranging from mild and transient side effects to rare and severe complications [16]. However, most of the AEFIs are preventable [17]. And It’s important to note that while these AEFI are concerning, the overall risk-benefit analysis continues to strongly support COVID-19 vaccination due to the substantial reduction in severe illness, hospitalizations, and deaths [18]. The Vaccine Adverse Event Reporting System (VAERS) is a national surveillance program in the United States that collects and analyzes reports of AEFI that occur after vaccination [19], [20]. VAERS helps to monitor the rise of recognized AEFI and identify possible patient factors contributing to specific AEFI [21]. It also offers its extensive database for research on AEFI.

In our previous study, we conducted a temporal analysis to investigate the patterns of AEFIs categorized by System Organ Classes (SOCs) sourcing from the VAERS database [22]. Our analysis revealed that symptoms falling under SOC 22 (general disorders and administration site conditions) were the most commonly reported adverse effects [23], [24]. Additionally in [25], we employed a logistic regression model to identify significant SOCs associated with age and gender. Our results indicated that females showed significantly higher odds in SOC 19 (related to Pregnancy, puerperium, and perinatal conditions), whereas males exhibited higher odds in SOC 25 (related to Surgical and medical procedures). Moreover, individuals over 65 years old demonstrated a higher probability of Cardiac disorders, while those aged 18–65 displayed greater susceptibility to Skin and subcutaneous tissue disorders. Pillay el. al (2022) synthesized evidence concerning the risk factors associated with myocarditis and pericarditis following the administration of COVID-19 bivalent vaccines [26]. They concluded that in the 18–29 age group, myocarditis incidence after Moderna vaccination is higher than with Pfizer. For men in this age range, extending the dosing interval to ≥ 56 days could significantly reduce myocarditis or pericarditis cases. Among adolescents and adults, over 90% of myocarditis instances affected men with symptoms appearing two to four days after the second dose. Unlike myocarditis, pericarditis exhibits greater variability in patient age, sex, onset timing, and hospital admission rates in reported cases. In 2023, Turabian conducted a longitudinal and prospective study of patients with 4th dose of bivalent mRNA vaccines [27]. He identified that Women, Socio-Health Care Workers, Chronic Diseases of the blood, Chronic Diseases of Endocrine, and Chronic Diseases of Circulatory system are the significant risk factors after the injection of COVID-19 bivalent vaccines.

In this study, we utilized the Medical Dictionary for Regulatory Activities (MedDRA), an internationally recognized medical terminology developed by the International Council for Harmonization of Technical Requirements for Pharmaceuticals for Human Use (ICH) [28]. After categorizing symptoms into 27 SOCs, we conducted an analysis using regression models and categorical analysis. Specifically, we determined the average number of symptoms per individual by categorizing cases based on sex, age groups, and vaccine manufacturer. Furthermore, we applied logistic regression to investigate the association between the occurrence of an SOC and individual factors such as sex, age, and vaccine manufacturer. To assess correlations among SOCs, we generated a rank-based correlation matrix.

## Methods

### 2.1. Data source

We collected adverse event reports post-COVID-19 bivalent vaccination, from 9/15/2022 to 9/1/2023 through the Vaccine Adverse Event Reporting System (VAERS). These reports are structured into three Comma-Separated-Value (CSV) files—VAERSDATA.CSV, VAERSVAX.CSV, and VAERSSYMPTOMS.CSV—categorized by year. VAERSDATA.CSV comprises demographic details, vaccination specifics, adverse event timing, symptom descriptions, allergy history, and serious outcomes. VAERSVAX.CSV offers data on vaccine types and manufacturers linked to each adverse event, while VAERSSYMPTOMS.CSV outlines symptoms associated with each event based on the Preferred Term (PT) in the MedDRA terminology. The primary key ‘VAERS_ID’ serves as the linking factor across the three tables.

In this project, we utilized MedDRA to characterize post-vaccination symptoms. MedDRA acts as a standardized vocabulary for AEFI reporting, ensuring uniform classification and analysis of safety data across diverse pharmaceutical products and clinical studies [28]. MedDRA’s Terminology follows a hierarchical structure with five levels: SOC, High Level Group Term (HLGT), High Level Term (HLT), PT, and the lowest Level Term (LLT). As of September 2021, MedDRA version 24.1 comprises over 84,000 PTs, employed by VAERS for coding and categorizing AEFI [29].

### 2.2. Reported symptoms mapped to MedDRA terminology

We applied a similar method to that of Li et al. for categorizing AEFI into their respective SOCs in this study [30]. Within VAERS, AEFI are reported through a voluntary and spontaneous system, with healthcare professionals, vaccine recipients, or concerned individuals supplying information on potential vaccine-related AEFI [31]. The reported events undergo categorization and coding using the MedDRA system, which organizes symptoms into SOCs [32]. Precisely, SOC (refer to Supplementary Table S1) serves as the highest-level hierarchical structure, broadly categorizing medical concepts based on factors like etiology, manifestation site, or purpose [7]. In this analysis, the reported events were harmonized with SOC classification, establishing a standardized framework for grouping related AEFI [32].

### 2.3. Statistical analysis

In this research, all analyses were conducted using RStudio with R version 4.3.1 and STATA version 17.0 SE-Standard Edition [34], [35]. Statistical significance was determined based on a two-tailed P-value of ≤ 0.05.

#### 2.3.1. Zero-truncated poisson regression

Given that each subject in our dataset reported at least one SOC, we employed a zero-truncated Poisson regression model for the data analysis [36]. The model was adjusted for sex, age (categorized into groups: (0–5), [5–12), [12–18), [18–65), 65+, and Unknown), and vaccine manufacturers (Pfizer and Moderna). This adjustment allowed us to estimate and compare the number of symptoms across various sex, age, and vaccine manufacturer groups.

#### 2.3.2. Logistic regression

Logistic regression analysis was carried out to investigate potential risk factors linked to each SOC, including gender, age, and vaccine manufacturer [37]. To streamline the analysis, the initial SOC count was converted into a binary outcome variable (1: SOC ≥ 1, 0: SOC = 0) in the logistic regression.

#### 2.3.3. Spearman rank correlation coefficient

Due to the significantly skewed and non-normally distributed nature of SOC counts, we chose to utilize Spearman’s rank correlation coefficient as a nonparametric metric for assessing correlations between SOCs [38]. This coefficient is suitable for non-normally distributed data and has proven effective in capturing the correlation between two variables. To illustrate the strength of pairwise correlations among SOCs, we generated a correlation matrix for the SOCs.

## Results

In our comprehensive analysis of adverse events following COVID-19 bivalent vaccine, Table 1 shows the descriptive statistics for this study.

**Figure 1:**
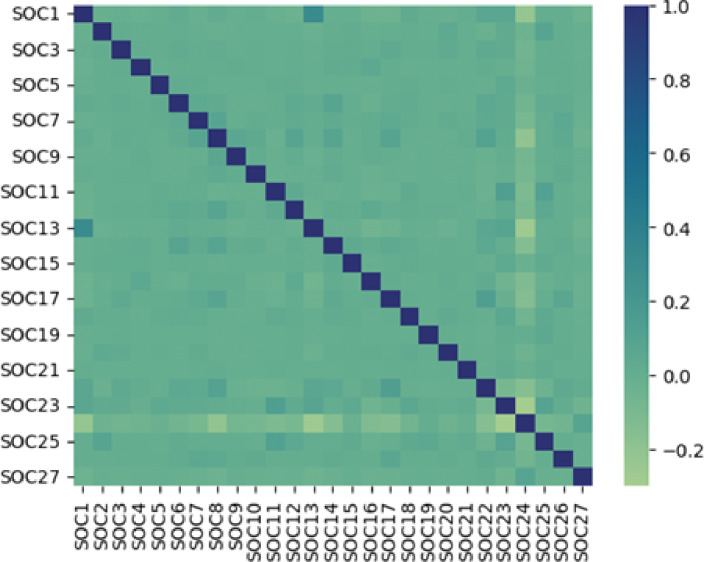
The pairwise correlation matrix of SOCs determined by Spearman’s method

We employed Spearman’s method [38] to compute the pairwise correlation matrix of SOCs, as illustrated in Fig. 1. The color and size of each data point signify the strength of the correlation between SOCs. To identify significant correlations, we applied a threshold of 0.2 or higher for the correlation coefficient [39], [40]. Our analysis revealed a substantial positive correlation between SOC 1 (Infections and infestations) and SOC 13 (Respiratory, thoracic and mediastinal disorders) (ρ = 0.3), along with a remarkable negative correlation between SOC 23 (Investigations) and SOC 24 (Injury, poisoning, and procedural complications) (ρ=−0.3), SOC 13 (Respiratory, thoracic and mediastinal disorders) and SOC 24 (Injury, poisoning and procedural complications)(ρ=−0.27), SOC 1(Infections and infestations) and SOC 24 (Injury, poisoning and procedural complications) (ρ=−0.22), SOC 8 (Nervous system disorders) and SOC 24 (Injury, poisoning and procedural complications)(ρ=−0.21).

The analysis conducted using a zero-truncated Poisson model yielded an estimated mean of 6.179 symptoms per individual in the study group, with a 95% confidence interval (CI) of (6.164, 6.195), a median of 6.82, an interquartile range (IQR) of (5.72, 7.11), a 2.5th percentile of 2.45, and a 97.5th percentile of 7.85. Upon adjusting for gender, age group, and vaccine manufacturer, the zero-truncated Poisson regression results highlighted variations in the number of symptoms based on sex, age groups, and vaccine manufacturers.

The study revealed that individuals aged 0 to 5 reported the fewest symptoms, averaging 2.27, while those aged over 65 reported the most, with an average of 7.13 symptoms. Moderna, the vaccine manufacturer associated with the higher symptom count, led with an average of approximately 6.27 symptoms, compared to 6.12 symptoms for Pfizer. Regarding gender differences, females reported a mean of approximately 6.81 symptoms, while males had a mean of 5.59. Individuals with an unknown gender typically averaged 3.4 symptoms.

The study revealed significant associations between 23 SOCs and age groups, excluding SOC 5 (Endocrine disorders), SOC 15 (Hepatobiliary disorders), SOC 21 (Congenital, familial and genetic disorders), and SOC 27 (Product issues). Additionally, it identified significant correlations between 24 SOCs and genders, excluding SOC 15 (Hepatobiliary disorders), SOC 19 (Pregnancy, puerperium and perinatal conditions), and SOC 21 (Congenital, familial and genetic disorders). Among these, 10 SOCs (SOC 4 (Immune system disorders), SOC 6 (Metabolism and nutrition disorders), SOC 11 (Cardiac disorders), SOC 12 (Vascular disorders), SOC 13 (Respiratory, thoracic and mediastinal disorders), SOC 16 (Skin and subcutaneous tissue disorders), SOC 17 (Musculoskeletal and connective tissue disorders), SOC 22 (General disorders and administration site conditions), SOC 25 (Surgical and medical procedures), SOC 27 (Product issues)) demonstrated statistically significant associations with vaccine manufacturers.

**Figure 2:**
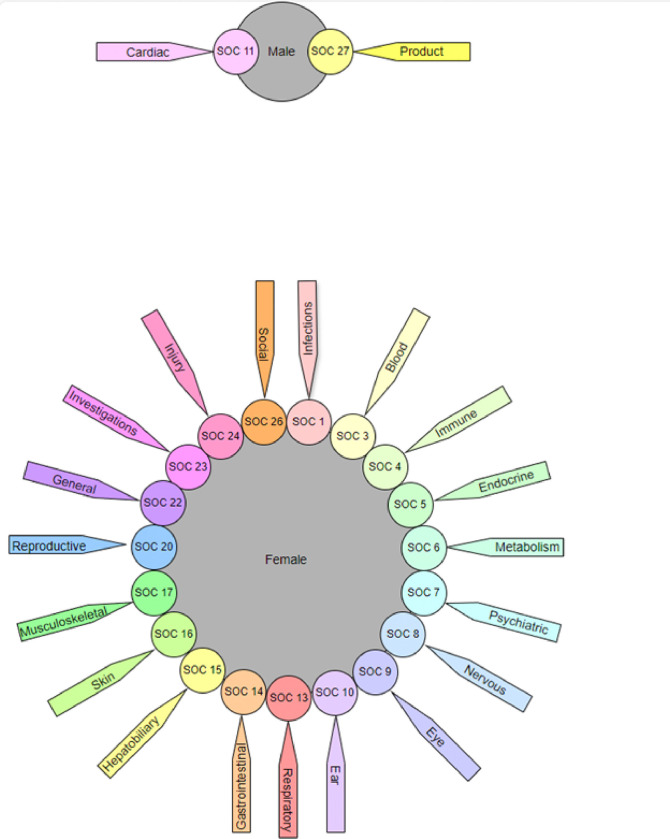
The significantly reported System Organ Classes associated with each gender

Figure 2 shows the significantly reported SOCs associated with each gender. It indicated that females exhibited a significantly higher likelihood than males in the majority of SOCs, except SOC 11 (Cardiac disorders) (OR = 0.84), and SOC 27 (Product issues) (OR = 0.67).

**Figure 3:**
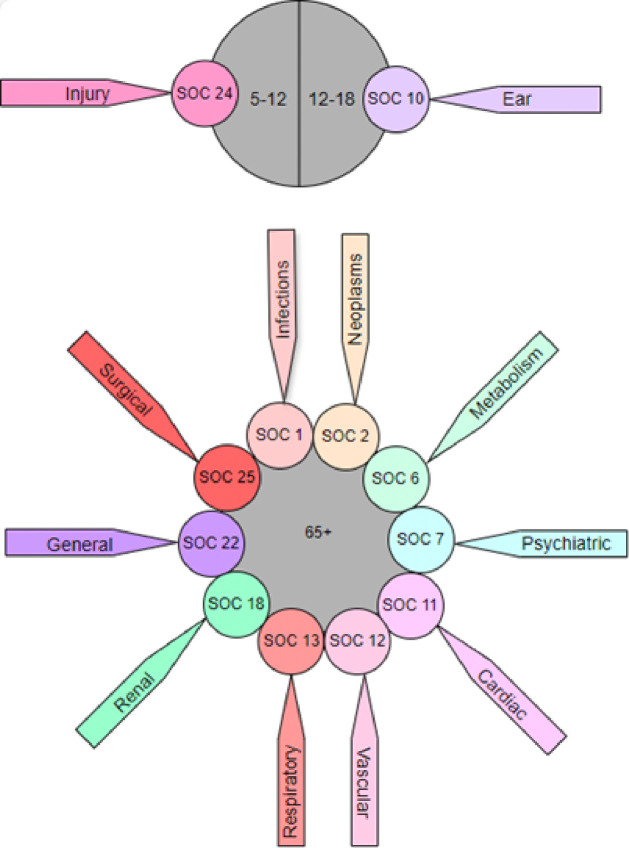
The significantly reported System Organ Classes associated with each age group

Figure 3 illustrates the significantly reported SOCs associated with each age group. Individuals aged 65 and above displayed a significantly higher likelihood of experiencing symptoms in various SOCs, including Infections and infestations (*P* < .001), Neoplasms benign, malignant and unspecified (including cysts and polyps) (*P* = .001), Metabolism and nutrition disorders (*P* < .001), Psychiatric disorders (*P* = .005), Cardiac disorders (*P* < .001), Vascular disorders (*P* < .001), Respiratory, thoracic and mediastinal disorders (*P* < .001), Renal and urinary disorders (*P* < .001), General disorders and administration site conditions (*P* < .001), Investigations (*P* < .001), Surgical and medical procedures (*P* < .001). In contrast, children aged 5–12 years old were more susceptible to experiencing symptoms in Injury, poisoning and procedural complications (*P* < .001), while those in the age group of 12–18 were more susceptible to experiencing symptoms related to SOC 10 (Ear and labyrinth disorders) (*P* < .001).

**Figure 4:**
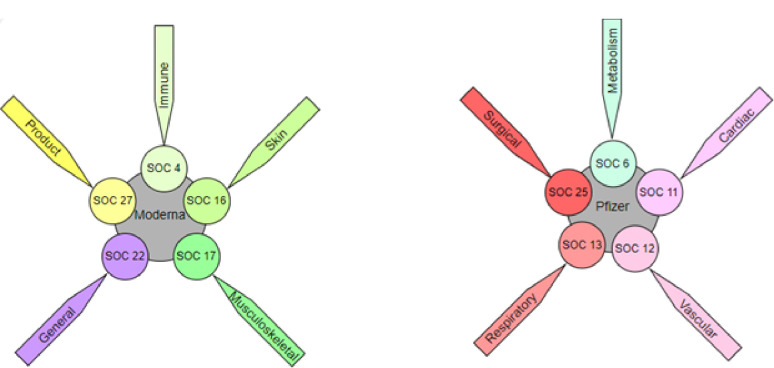
The significantly reported System Organ Classes associated with each vaccine manufacturer

Figure 4 shows the significantly reported SOCs associated with each vaccine manufacturer. Moderna significantly showed the higher odds in SOC 4 (Immune system disorders) (*P* < .001), SOC 16 (Skin and subcutaneous tissue disorders) (*P* < .001), SOC 17 (Musculoskeletal and connective tissue disorders) (*P* < .001), SOC 22 (General disorders and administration site conditions) (*P* < .001), and SOC 27 (Product issues) (*P* < .001), while Pfizer significantly demonstrated the higher odds in SOC 6 (Metabolism and nutrition disorders) (*P* < .001), SOC 11 (Cardiac disorders) (*P* < .001), SOC 12 (Vascular disorders) (*P* = .001), SOC 13 (Respiratory, thoracic and mediastinal disorders) (*P* = .005), and SOC 25 (Surgical and medical procedures) (*P* < .001). Last but not least, in the analysis of all 27 logistic regression tests, the findings consistently indicated a high degree of statistical significance, with *P* < .001, except for the logistic regression test related to SOC 21 (Congenital, familial, and genetic disorders).

## Discussion

The analysis of correlations yielded intriguing findings. We noted a strong positive correlation between SOC 1 (Infections and infestations) and SOC 13 (Respiratory, thoracic and mediastinal disorders) (ρ = 0.3), which can be elucidated by various interconnected factors. Biologically, Infections, especially respiratory infections, can directly impact the respiratory system [41]. On the other hand, pathogens like viruses or bacteria, commonly and synergistically target the respiratory tract, directly influencing the development of disorders within the respiratory and thoracic system [42]. Secondly, complications arising from respiratory infections may exacerbate pre-existing respiratory conditions [43], [44]. For example, a respiratory infection may worsen conditions like asthma or chronic obstructive pulmonary disease (COPD). Diagnostic overlap, where the diagnostic criteria for infections and respiratory/thoracic disorders may have an intersection, further contributes to the observed correlation [45]. Some symptoms, such as coughing or difficulty breathing, can be indicative of both infectious and non-infectious respiratory issues, leading to a correlation in reported cases. Additionally, infections often trigger an inflammatory response, and inflammation is a common feature in many respiratory disorders [46]. This shared inflammatory response could also account for the observed correlation. We also observed a strong negative correlation between SOC 23 (Investigations) and SOC 24 (Injury, poisoning, and procedural complications), suggesting that stringent safety measures and thorough investigations in place for COVID-19 bivalent vaccines may contribute to reducing the likelihood of procedural complications and injuries.

The correlation results from the COVID-19 vaccines revealed notable similarities in the associations between age groups, genders, and vaccine manufacturers with SOCs [25]. For instance, both datasets indicated that individuals aged 65 and above were more likely to experience symptoms in various categories, including Infections and infestations, Neoplasms(benign, malignant and unspecified (including cysts and polyps), Metabolism and nutrition disorders, Psychiatric disorders, Cardiac disorders, Respiratory, thoracic and mediastinal disorders, Renal and urinary disorders, General disorders and administration site conditions, Investigations, and Surgical and medical procedures. Additionally, both vaccines highlighted that children aged 5–12 were more susceptible to symptoms related to Injury, poisoning, and procedural complications. These consistent age-specific associations underscore the importance of conducting tailored safety assessments based on the unique characteristics of each vaccine.

The comparison between the COVID-19 vaccines and COVID-19 bivalent vaccines reveals distinct patterns in symptomatology. While both vaccines exhibit a positive correlation between age and the likelihood of experiencing symptoms, significant differences emerge in the severity of symptoms reported. For COVID-19 vaccines, the mean number of symptoms per individual is 4.243, with individuals aged 5 to 12 reporting the fewest symptoms (average of 2.43) and those aged 18 to 65 reporting the most (average of 4.52) [25]. In contrast, COVID-19 bivalent vaccines present a higher symptom burden, with an estimated mean of 6.179 symptoms per individual. The differences in symptom profiles highlight the vaccine-specific effects on symptomatology. Examining and understanding these distinctions is crucial for tailoring safety considerations among individuals receiving different COVID-19 vaccines.

Our correlation study exhibits several notable strengths. Firstly, in contrast to other investigations, our analysis encompasses VAERS data spanning over two years. A larger dataset enhances correlation accuracy and reliability, providing statistical power to detect meaningful relationships and explore variations in diverse subgroups or conditions. Additionally, our study meticulously examined the impact of individual factors, including gender, age group, and vaccine manufacturers. This detailed analysis, conducted separately for each factor, enabled the identification of subpopulations more susceptible to adverse events following COVID-19 bivalent vaccination. Lastly, we applied widely recognized statistical methods to scrutinize the correlation between SOCs. Our methodological approach not only allowed for the identification of the most prevalent adverse events associated with COVID-19 bivalent vaccines but also provided insights into their underlying mechanisms. This valuable information has the potential to assist healthcare providers in the more effective management and treatment of adverse events linked to COVID-19 bivalent vaccines.

However, it is essential to recognize the limitations of our study. Firstly, VAERS serves as a system for signaling potential AEFI, and its reliance on voluntary self-reporting introduces the potential for reporting bias, challenging the establishment of direct causal relationships [47]. For instance, the negative correlation observed in our analysis might be influenced by the reporting dynamics, where an increased focus on investigations following vaccination could lead to a tendency to report fewer injuries, poisonings, or procedural complications, resulting in a negative correlation. Additionally, statistical correlations can arise due to random variation in a specific dataset, and the observed correlation might not necessarily imply a causal relationship. Further investigation and analysis are required to establish causation and provide a comprehensive understanding of the findings. Furthermore, the study’s interpretive limitations arise from the absence of personal health information, such as body mass index (BMI) and medical history, with only sex, age, and vaccine manufacturer available for the study population.

## Conclusion

In this study, we categorized post-vaccination symptoms into 27 System Organ Classes (SOCs) using VAERS data and applied advanced statistical methods to analyze the occurrence of these symptoms across different study subgroups.

Our research provides valuable insights into the relationships between symptoms, demographic factors, and risk factors associated with COVID-19 bivalent vaccination. This work enhances our understanding of vaccine safety, supporting the development of evidence-based vaccination strategies and targeted healthcare interventions. The findings also lay the groundwork for future studies, including case-control investigations, aimed at refining safety measures and improving vaccine outcomes for diverse populations.

## Figures and Tables

**Table 1:** Descriptive statistics for VAERS reports related to COVID-19 bivalent vaccination in the study

Total	
	**37,441**
**Sex**	
Male (%)	14,287 (38.16%)
Female (%)	21,333 (56.98%)
Unknown (%)	1,821 (4.86%)
**Age**	
(0–5) (%)	1,287 (3.44%)
[5,12) (%)	1,946 (5.20%)
[12,18) (%)	1,293 (3.45%)
[18–65) (%)	16,333 (43.62%)
65+ (%)	14,664 (39.17%)
Unknown (%)	1,918 (5.12%)
**Manufacturer**	
Pfizer\Biotech (%)	21,738 (58.06%)
Moderna (%)	15,703 (41.94%)
